# Rainfall decrease and red deer rutting behaviour: Weaker and delayed rutting activity though higher opportunity for sexual selection

**DOI:** 10.1371/journal.pone.0244802

**Published:** 2021-01-20

**Authors:** Marina F. Millán, Juan Carranza, Javier Pérez-González, Juliana Valencia, Jerónimo Torres-Porras, Jose M. Seoane, Eva de la Peña, Susana Alarcos, Cristina B. Sánchez-Prieto, Leticia Castillo, Antonio Flores, Alberto Membrillo

**Affiliations:** 1 Wildlife Research Unit (UIRCP), Universidad de Córdoba, Córdoba, Spain; 2 Biology and Ethology Unit, Universidad de Extremadura, Cáceres, Spain; 3 Didáctica de las Ciencias Experimentales, Facultad de Ciencias de la Educación, Universidad de Málaga, Málaga, Spain; 4 Department of Social and Experimental Sciences Teaching, Faculty of Educational Sciences, Universidad de Córdoba, Córdoba, Spain; Universita degli Studi di Sassari, ITALY

## Abstract

In the last decades, climate change has caused an increase in mean temperatures and a reduction in average rainfall in southern Europe, which is expected to reduce resource availability for herbivores. Resource availability can influence animals' physical condition and population growth. However, much less is known on its effects on reproductive performance and sexual selection. In this study, we assessed the impact of three environmental factors related to climate change (rainfall, temperature and vegetation index) on Iberian red deer *Cervus elaphus hispanicus* reproductive timing and sexual behaviour, and their effects on the opportunity for sexual selection in the population. We measured rutting phenology as rut peak date, the intensity of male rutting activity as roaring rate, and the opportunity for sexual selection from the distribution of females among harem holding males in Doñana Biological Reserve (Southwest Spain), from data of daily observations collected during the rut over a period of 25 years. For this study period, we found a trend for less raining and hence poorer environmental conditions, which associated with delayed rutting season and decreased rutting intensity, but that appeared to favour a higher degree of polygyny and opportunity for sexual selection, all these relationships being modulated by population density and sex ratio. This study highlights how climate change (mainly rainfall reduction in this area) can alter the conditions for mating and the opportunity for sexual selection in a large terrestrial mammal.

## Introduction

Environmental factors strongly influence many elements of ecology and behaviour of organisms, including reproductive strategies and associated selective processes [1–7]. In the last decades, human induced climate change has been shown to alter phenology, spatial distribution, and many life history traits in several plants, birds, amphibians or terrestrial mammals [3, 8–18]. However, the effects of climate change are likely to be diverse depending on geographical areas and on the ability of species to track phenological variations [19, 20].

The red deer *Cervus elaphus* is a large mammal broadly distributed throughout Eurasia. For this species, the effects of climate change could be very different in northern and southern populations. For instance, previous work on red deer populations in Norway found that earlier and warmer springs resulted in a longer period of high-quality resource availability [21]. However, other studies have shown significant disadvantages for red deer in high latitudes due to climate warming. A trend for red deer and other ungulates to reduce body size and fecundity in response to increasingly warm winters has been reported in Northern Europe [22, 23]. Regarding reproductive behaviour, red deer populations have been observed to shift breeding phenology in some Central (France) and Northern (Norway) European populations in response to climate variations. However, phenological responses may be diverse. For instance, it has been found a delay in red deer female ovulation in response to shorter summers in Norway [24], while red deer and reindeer breeding phenology in the Isle of Rum (Scotland) and North Finland, respectively, advanced [25, 26]. Also, other authors found later calving and rutting times in Norway compared to France, related to the late onset of plant phenology [27]. In the case of Mediterranean habitats of Southern Europe, very little is known about how climate change may affect red deer reproductive processes [28].

In warm regions of Southern Europe, climate change is expected to produce an increase in average temperatures and evaporation and a greater differentiation between winter and summer, which means an increase in drought events [29–33].

The mating season of Iberian red deer (*C*. *e*. *hispanicus)* in Southern Spain takes place at the end of summer, in September, within the period of lowest food availability. In this region, summer is a severe dry and hot season, when the availability of forage is minimal, in contrast to other regions in northern Europe where the limiting season is winter because of the cold temperatures and the snow [34–36]. Drought events reduce water availability for plant growth and, hence, forage for herbivores, thus affecting their growth and reproduction rates [37–39].

Forage availability has been shown to have important consequences on mating strategies, reproductive success and sexual selection [40, 41]. For red deer, resource (vegetation) distribution determines female dispersion and consequently the mating strategies used by males [42, 43]. Also, previous work has found that under conditions of resource scarcity, females aggregated in the few available patches of food, and other males gathered around, so that harem size increased until harem holders were no longer able to monopolize those big harems [44]. This female clumping increased the variance in male mating success, which influences the opportunity for sexual selection [45, 46].

Furthermore, male mating success depends on body condition and the development of secondary sexual characters involved in reproduction, such as antler size, which are also determined by environmental conditions [47–50]. For instance, it has been found a higher investment in red deer antler development relative to body weight under favourable environmental conditions [51]. A reduction in red deer body and antler size related to drought periods has been reported in Southern Spain as well [52].

In Doñana National Park (Southwestern Spain) red deer typically aggregated during the mating season in an ecotone between the shrub and the marsh zone [53], and some males adopt a territorial mating strategy instead of the most common strategy for this species based on harem defense [54]. In this area, males often establish territories in better forage areas or in paths used by females to get to these areas, as a strategy to contact and get access to females [54, 55]. Females using other nearby areas before the rut also tend to aggregate at these mating areas just during the mating season and join harems, probably to reduce sexual harassment by bachelor males [42].

In the last decades, Doñana has suffered an important increase in aridity and seasonality due to climate warming [56–61]. In addition, the increase of water extraction from aquifers for urban and agricultural use has also contributed to aridity and habitat fragmentation [62–67]. Thus, we expected the increasing drought events in Doñana to reduce resource availability, which may limit red deer energy intake and body condition for reproduction, and therefore, may delay female ovulation and reduce the intensity of male sexual activity and rutting behaviour [28, 68]. All these changes might have concominant effects on the degree of polygyny and the opportunity for sexual selection in the population [45, 46].

Therefore, the aim of this study was to assess the impact of climate variations on some features of red deer mating behaviour: 1) rut phenology; 2) the intensity of rutting activity; and 3) their consequences on the opportunity for sexual selection in the population in Doñana National Park. To do so, we used data on deer presence and behaviour, including roaring phenology and intensity, registered from daily observations during the rut over a 25-years period, and their corresponding estimations of opportunity for sexual selection, along with main environmental variables related to climate change. This study highlights how climate change can alter the conditions for mating and the opportunity for sexual selection in red deer populations.

## Methods

### Study area

This work does not have an implication in animal welfare since the data has been obtained through remote observations of the animals, without any interference or contact with them. The study was carried out in Doñana Biological Reserve, a protected research area of 6,794 ha within the Doñana National Park (Southwest of the Iberian Peninsula, ca 37°10’N, 6°23’W). Doñana is mainly characterized by a seasonal marshland limiting with a shrub zone. Our observation area included four fixed positions (observation points) located in an ecotone of open meadows extended along the border between the shrub and the marsh. Each observation point covered an area of 70 ha, between 100 to 700 m apart from the nearest neighbouring point. Their locations were chosen based on the areas where deer are typically present during the mating season. Deer using the area under each observation point were considered independent on the basis of the known deer movements in the area [42, 69].

Doñana has a Mediterranean climate, with mild wet winters and hot dry summers. The average annual rainfall is 549 mm and the rainy seasons are autumn and spring. The rut occurs just after the season of resource scarcity and deer gather in those meadows with some remaining pasture patches [53].

### Data collection

The data used in this study were collected over a period of 25 years (1995–2019). Data were based on daily observations during the rut, typically from September 1 to 24, carried out during the three hours before dusk. At the end of the daily observation time, all adult males, females, subadult and young males were registered and their positions located on a map of the area under observation. Population density was annualy estimated as the daily mean number of total deer recorded in the four observation points in the study area during the rutting period. Every day at dusk, we registered the number of roars audible from each observation point. Listening was conducted twice with one-minute duration each, with a lapse between them of at least one minute without recording. We used the mean number of roars per minute as the daily intensity of roaring. This measure has been previously used as an indicator of the stage of the rut [44] and roaring in red deer has been demonstrated to relate to fighting ability and reproductive success [70, 71]. The day with the highest roaring rate of each year was considered the peak day of the rut.

Adult males were individually identified by the shape of their antlers. Individuals were identified annually since antlers were cast every year and deer in the area were not marked. We used the focal group sampling method [72] to observe the sexual behaviour of the adult males present within the area covered from each observation point. Those females that were with a male until the end of the day, either within his territory or grouped with him, were considered as his harem for that day [54].

### Opportunity for sexual selection

For each year, we calculated the opportunity for sexual selection at the peak day of the rut by the I_mates_ parameter [73]:

$$I_{mates}=\frac{R(H-R)+(RV_{harem}/H)}{R^{2}}$$

where R is the sex ratio as number of females divided by the number of males [74], H is the mean harem size of mating males (only those males with a harem), and V_harem_ is the variance in harem size among mating males. To estimate R, we included all the adult males, regardless they had a harem or not, and all those females that were included in harems kept by a male, i.e., those females affecting the distribution of mates among males, and hence I_mates_.

### Environmental variables

We collected data of the seasonal rainfall and temperature in Doñana for the entire study period from the Singular Scientific-Technical Infrastructure of Doñana Biological Reserve database.

The Normalized Difference Vegetation Index (NDVI) has been previously used in several studies of global change and forage availability for large herbivores in open landscapes [21, 75–77]. We calculated the NDVI values of each year from 450 points distributed throughout the study area to obtain a consistent NDVI value. The NDVI values were obtained from Landsat images courtesy of the U.S. Geological Survey. These images have a resolution of 30 m/pixel, so the NDVI value of each coordinate corresponded to an area of 900 m2. The NDVI calculation was conducted in QGIS [78] according to the expression:

$$NDVI=\frac{\left(NIR-VIS\right)}{\left(NIR+VIS\right)}$$

where NIR is the near-infrared reflectance and VIS is the visible red.

We discarded images with high cloudiness and applied an atmospheric correction before calculating the NDVI values. We used the SCP complement (Semi-Automatic Classification Plugin) to apply the DOS atmospheric correction [79, 80], which is one of the most commonly used atmospheric correction methods.

To include NDVI data of our whole study period, we had to use Landsat 7 images from 2000 to 2019, and Landsat 4–5 images from 1995 to 1999, because of the non-availability of images from the same satellite for the whole period.

### Statistical analysis

We used the “*sliding window*” analysis in the package *‘climwin’* in R [81, 82] to select the time windows for the environmental variables temperature, rainfall and NDVI, that most affected our response variables roaring rate, rut peak date and opportunity for sexual selection (I_mates_). We used monthly intervals of mean rainfall and temperature in the same analysis, and NDVI in another aside because of the difference in the continuity of the data between the variables. We chose the most relevant time windows from one year before the rut and adjusted “relative type” [81] time windows to account for differences in the annual rutting date. Then, we used the “*randwin”* function in ‘*climwin’* [81] to run 1000 randomizations for each sliding window analysis to ensure that the weather signals found were not false positive results and obtained a p-value to confirm the reliability of these climate windows. Sliding window analysis has been previosuly used in determining the effect of weather variables on red deer breeding phenology [83].

We built Generalized Linear Mixed Models (GLMM) and Linear Models (LM) to explore the effect of these environmental variables, in their selected temporary windows, along with other population variables on the three response variables. The fixed effects tested were total rainfall, mean temperature and NDVI from the temporary windows above, mean population density during the rut, and operational sex ratio at the rut peak day (OSR: defined as the ratio of sexually active males to females [84]). Observation point nested to year was fitted as random term, except in the model for rut peak, because of the singularity of the model when adding this random factor. Model selection to identify the main drivers on our dependent variables, was carried out by backward elimination based on p-values in favour of information thery approaches like ∆AIC or BIF [85–88]. We first fitted full models with all the study variables and their double interactions and then removed the non-significant terms, one at a time, following the principle of marginality [86]. Because of the non-normality of the variables roaring rate and I_mates_, we fitted two GLMMs (for roaring rate and I_mates_) with a Poisson distribution [89], since our variables were calculated from counting data, and a single linear model for the variable rut peak date. The presence of overdispersion was checked in the three models. We represented the variance of the model explained by the fixed effects by using adjusted R^2^, marginal R^2^ and conditional R^2^ for the variance explained by fixed and random effects [90]. We found correlations between the variables rainfall and temperature (for rut peak: r = 0.65, p < 0.001; for roaring rate: r = 0.62, p < 0.001; for I_mates_: r = 0.23, p = 0.02), so we used the VIF factor (variance inflation factor) [74] to check if both variables could be included in the same model as fixed effects. In the three models we obtained VIF values < 3 for all the variables so both rainfall and temperature were maintained as explanatory variables [91, 92]. We plotted the variation of the environmental variables along the study years to check its trend over time. All statistical analyses were conducted in R software [93].

## Results

**Fig 1:**
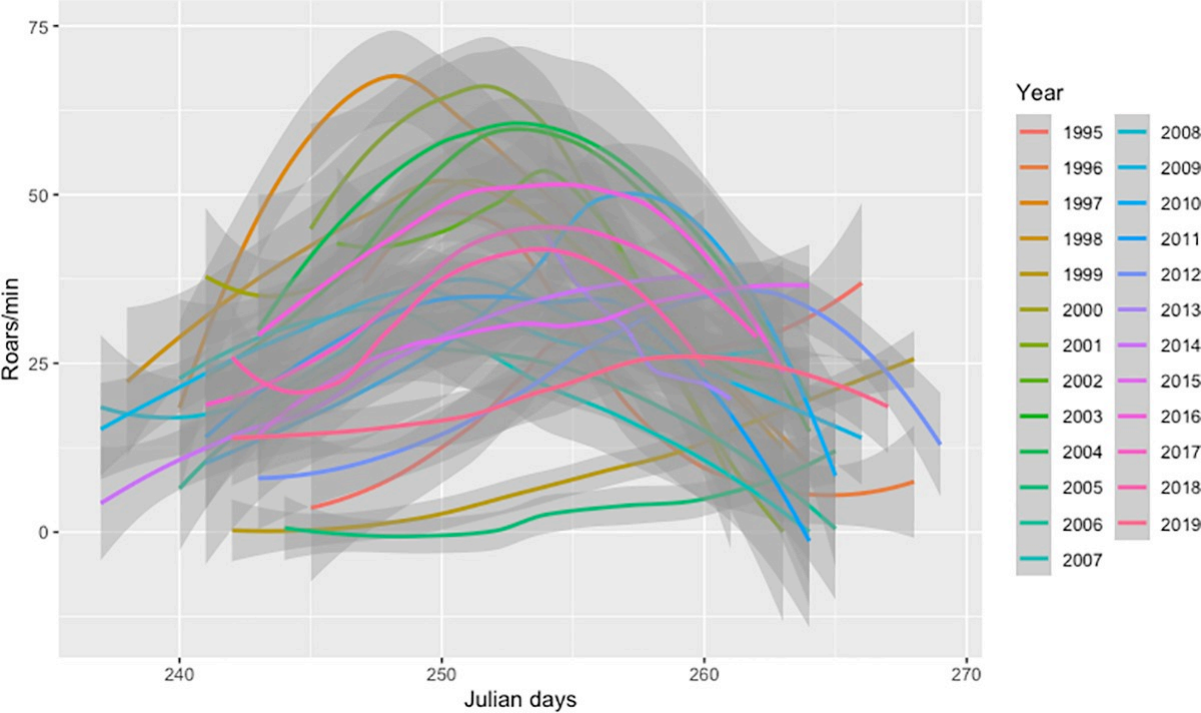
Variation of the roaring rate (roars/min) along the rut period in Julian days. Colours indicate different study years and grey shadows represent confidence intervals (95%). Julian day 250 corresponds to September 7^th^.

The pattern of variation of roaring rate along the rut showed a similar concave shape for most years, although both the maximum value reached each year and the date when it occurred (i.e, rut peak date) were variable (Fig 1).

Sliding window analysis showed that for rainfall, data from autumn to winter was the most influential for the three study variables, with a negative effect on the rut peak date and I_mates_, and a positive one on roaring rate (Table 1). The effect of temperature in winter was the most important for the rut peak date and the roaring rate, it being negative and positive respectively, while for I_mates_ October temperature seemed to be the most relevant temperature period. NDVI at the end of spring and at summer had the greatest effect, negative for the rut peak and I_mates_ and positive for roaring rate (Table 1).

**Table 1:** Temporary windows of the environmental variables chosen following the sliding window analysis for the response variables rut peak, roaring rate and opportunity for sexual selection (I_mates_) in the next September rutting season. The estimated effect of each climatic-factor window on the biological response variable is shown, with standard error (SE) ant a *t* value. P_E_ is the p-value for the estimated linear effect of each climatic-variable window on the response variables, and P_W_ is the p-value for window selection (obtained after 1000 randomizations).

Response	Environmental variable	Temporary window	Estimate	SE	t value	P_E_	P_W_
	Rainfall	October-December	-1.959	0.348	-5.627	< 0.001	< 0.01
Rut peak	Temperature	December-March	-1.999	0.505	-3.959	< 0.001	< 0.01
	NDVI	May-August	-47.782	10.432	-4.580	< 0.001	< 0.01
	Rainfall	September-December	0.220	0.019	11.890	< 0.001	< 0.001
Roaring rate	Temperature	January-February	0.171	0.015	11.471	< 0.001	< 0.001
	NDVI	May-June	1.321	0.315	4.191	< 0.001	< 0.001
	Rainfall	November	-0.176	0.036	-4.926	< 0.001	0.03
I_mates_	Temperature	October	-0.244	0.054	-4.492	< 0.001	0.07
	NDVI	June-July	-4.364	0.929	-4.695	< 0.001	0.03

The best climate windows chosen were supported by the significant p-values obtained after 1000 randomizations. For the selected climate windows affecting the biological variable rut peak date, we obtained p-values < 0.01, for roaring rate p < 0.001, and for the opportunity for sexual selection p = 0.03 for both rainfall and NDVI windows, and only p = 0.07 for the October temperature window, meaning that selection of this window is not so strongly supported (Table 1).

Results of the linear model for the dependent variable rut peak date (Table 2) showed a negative effect of total rainfall from October to December, it being the only significant effect in the model, i.e., the more rain in autumn the earlier the next rutting season. The variance explained by the fixed factors was 24.2% (*R*^*2*^*)*.

**Table 2:** Linear model for the response variable rut peak (a date in September) and the fixed factors rainfall (previous year October-December), temperature (previous December-March), NDVI (previous May-August), population density and Operational Sex Ratio (OSR).

		Estimate	SE	*Z*	*p*
Fixed effects					
	Intercept	254.929	0.550	463.818	< 0.001
	Rainfall (Oct-Dec)	-2.164	0.925	-2.338	0.023
	Temperature (Dec-Mar)	-0.900	0.762	-1.181	0.242
	NDVI (May-Aug)	0.411	0.751	0.547	0.587
	Population density	-0.276	0.582	-0.474	0.64
	OSR	-0.527	0.540	-0.976	0.333
*Adjusted R*^*2*^		0.242			

For the response variable roaring rate (Table 3) we found a positive significant effect of total rainfall from September to December. Mean temperature from January to February showed a marginally significant, positive relationship with roaring rate. Operational sex ratio (OSR) and population density also showed strong positive effects. The effect of the NDVI was not significant by itself, but the interaction with the variable temperature was. We graphically explored this interaction, which showed that the positive effect of winter temperature on roaring activity was lost when NDVI in May and June was high, since with high NDVI values in May-June roaring rate tended to be always high regardless winter temperature. The fixed effects explained 57.3% of the variance of the data (R^2^_LMM(m)_)_,_ and the total variance explained by fixed and random effects was 85.5% (R^2^_LMM(c)_)_._

**Table 3:** Coefficients of the linear mixed-effects model on maximum annual roaring rate. *R*^2^_LMM(m)_ is the marginal R-squared and *R*^2^_LMM(c)_ is the conditional R-squared.

			Variance		SD
Random effects					
	Observation point: Year		0.034		0.186
		Estimate	SE	*Z*	*p*
Fixed effects					
	Intercept	4.067	0.035	116.592	< 0.001
	Rainfall (Sep-Dec)	0.093	0.045	2.058	0.040
	Temperature (Jan-Feb)	0.080	0.043	1.888	0.059
	NDVI (May-Jun)	0.024	0.038	0.631	0.528
	Population density	0.122	0.043	2.841	< 0.01
	OSR	0.154	0.058	2.677	< 0.01
	Temperature*NDVI	-0.123	0.034	-3.648	< 0.001
*R*^*2*^_LMM(m)_		0.573			
*R*^*2*^_LMM(c)_		0.855			

In the model for the response variable opportunity for sexual selection (Table 4), the OSR also had the greatest and positive effect, and the three environmental variables, total rainfall in November, mean temperature in October and NDVI in May and June, had negative significant effects. Population density effect was not significant in this model. The fixed effects explained 34.9% of the variance of the data (R^2^_LMM(m)_)_,_ and the total variance explained by fixed and random effects was 86.5% (R^2^_LMM(c)_)_._

**Table 4:** Coefficients of the linear mixed-effects model on the opportunity for sexual selection at rut peak. *R*^2^_LMM(m)_ is the marginal R-squared and *R*^2^_LMM(c)_ is the conditional R-squared.

			Variance		SD
Random effects					
	Observation point: Year		0.131		0.361
		Estimate	SE	*Z*	*p*
Fixed effects					
	Intercept	3.291	0.057	57.883	< 0.001
	Rainfall (Nov)	-0.114	0.050	-2.284	0.022
	Temperature (Oct)	-0.147	0.049	-2.997	< 0.01
	NDVI (Jun-Jul)	-0.177	0.062	-2.846	< 0.01
	Population density	0.052	0.062	0.848	0.397
	OSR	0.314	0.105	2.990	<0.01
*R*^*2*^_LMM(m)_		0.349			
*R*^*2*^_LMM(c)_		0.865			

**Fig 2:**
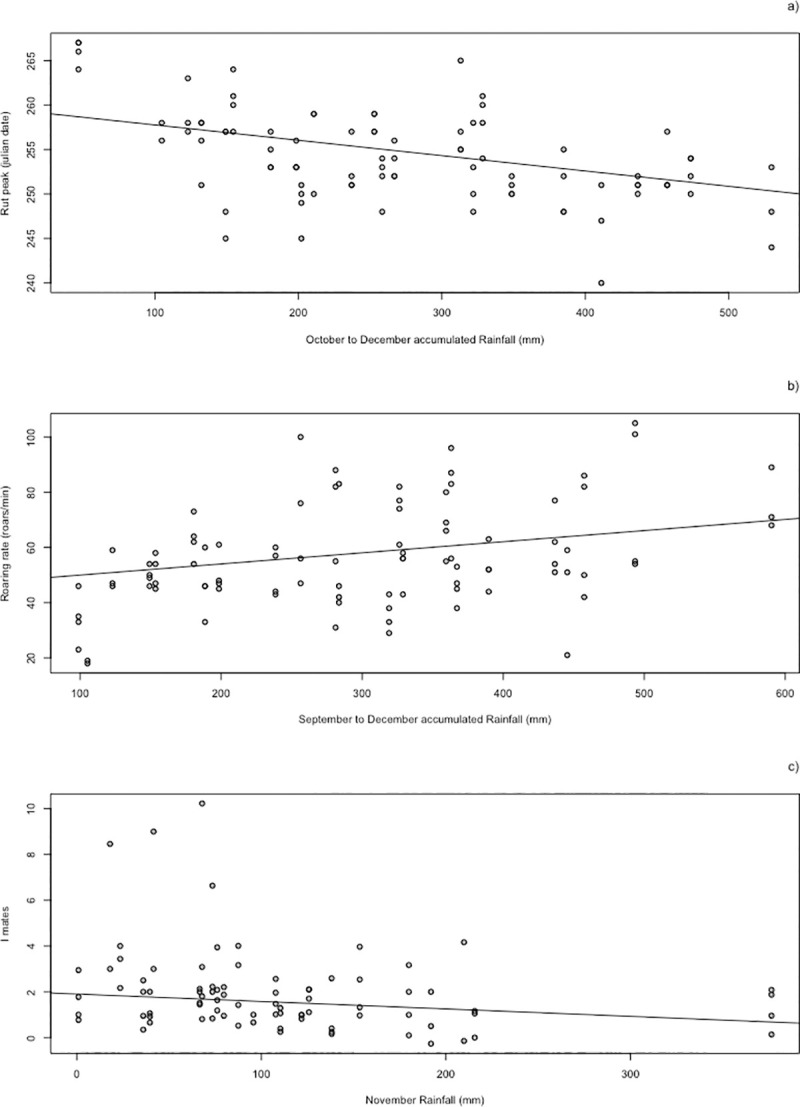
Predictions of the response variables rut peak (a), roaring rate (b) and opportunity for sexual selection (c) against their most relevant rainfall window in millimeters (filled black points and regression line). Raw data are also shown as open points.

**Fig 3:**
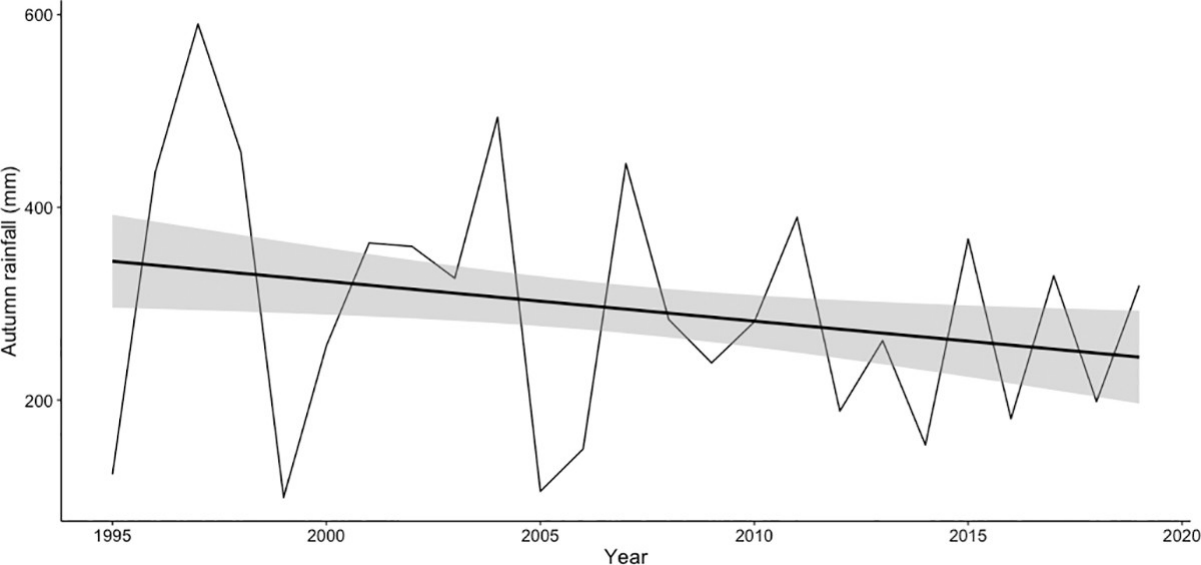
Variation of autumn rainfall in milimeters (September-December) over the study period. Points represent actual data and line represents the linear regression.

Autumn rainfall had an important effect in the three models and seemed to explain a certain amount of the variation of the response variables (Fig 2). Moreover, rainfall was highly variable between years but it showed an overall negative and significant trend over the study period (estimate = -4.146, SE = 3. 1.732, p = 0.019; Fig 3).

**Fig 4:**
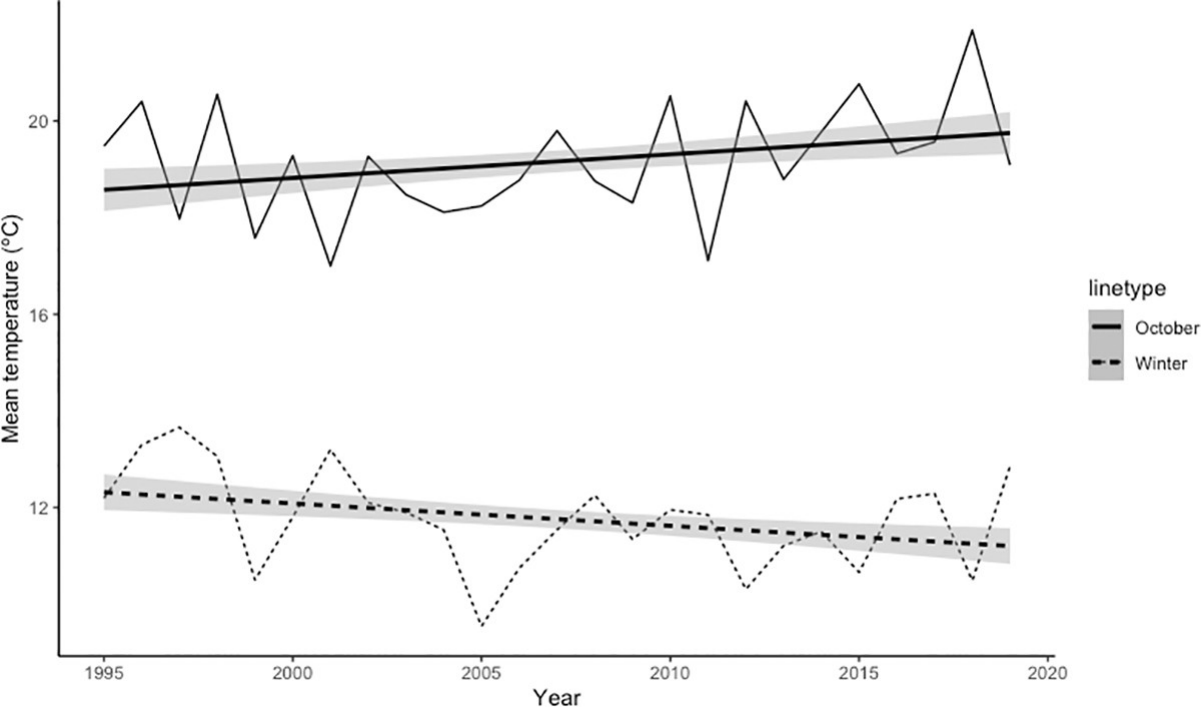
Temporary trend of the mean temperature (C°) in winter (dashed lines, from December to March) and in October (continuous lines). Straight lines represent the significant linear regressions with ± 2SD.

Mean temperature also varied differently depending on the season. Temperature in winter showed a negative significant trend over the years (estimate = -0.046, SD = 0.013, p < 0.001), while mean temperature in October had an increasing trend (estimate = 0.049, SD = 0.015, p < 0.01) (Fig 4). NDVI from May to August did not show a clear pattern of change along the years of study (estimate = -1.428 x 10^−5^, SD = 8.513 x 10^−4^, p = 0.987).

## Discussion

Variations in roaring intensity and rutting phenology over the 25 years of study suggest the influence of ambient and population factors producing pluriannual trends, which deserve attention to identify their causes and to the eventual prevention of their consequences. Our analyses have identified significant effects of three environmental variables (rainfall, temperature and NDVI) on the rut intensity and phenology, and on the opportunity for sexual selection in the population.

Although we did not measure reproductive success of males to compute actual opportunity for selection, but rather the number of females with them, our estimate of opportunity for sexual selection is based on Wade and Shuster method [73], who showed that the distribution of females among males was an accurate estimation of the opportunity for sexual selection in resource-defence based polygynous mating systems. Moreover, in red deer, the distribution of females among rutting males has been shown to be a good proxy of actual variance in reproductive success for red deer males [94, 95]. And also, for a number of Iberian red deer populations in Southern Spain, it has been previously seen that variations in the distribution of females per male were related to differences in the genetic estimation of polygyny among populations [44].

### Time windows for the environmental factors

Our analyses to find the most relevant time windows for the environmental variables, indicated that the occurrence of an early rutting season in September and a high roaring rate, were mainly determined by rainfall and temperature during the previous autumn and winter, as well as by NDVI values during the previous spring and summer. We interpreted these results as an effect of environmental variables on total resource availability and the recovery of the body condition that individuals need to face the next rutting season [96–98]. For instance, autumn rains affected the availability of biomass and grass in late spring and early summer, and therefore, they may influence deer body condition for reproduction [99, 100]. Additionally, warmer temperatures in winter favour the advance of spring vegetation growing, thus allowing deer to early recover their physical condition, and therefore, to reach a good energy status to reproduce [25]. The effect of high spring and summer NDVI values advancing the rut and increasing the roaring rate is consistent with the results for rainfall and temperature, since higher NDVI values, as an approximation of grass abundance, may mean higher resource availability for red deer.

For the opportunity for sexual selection, we found that it was mainly influenced (negative effects) by mean rainfall in November of the previous year, temperature in previous October and NDVI in June to July of the current year. Although we obtained a p-value for the temperature window of 0.07 for October, which indicated no clear selection for the most relevant time window affecting the opportunity for sexual selection, the choice of this window is in agreement with other climate variables that pointed to the importance of weather during the previous autumn and winter.

The most important time window for NDVI values appeared to be May-to-June, i.e. late spring, affecting positively the roaring rate, June to July affecting negatively the opportunity for sexual selection, and May-to-August affecting negatively (advancing) the rut peak date. These relationships are consistent with the results for rainfall and temperature, since higher NDVI value, as an approximation of grass abundance, may mean higher resource availability for red deer. Also, the negative relationship of NDVI and other environmental variables with the opportunity for sexual selection points to a stronger selection in those years when deer are in poorer condition.

### Models for the environmental and populational effects on the mating system

Linear models with the above environmental variables, in their time windows, along with population density and OSR as independent factors, for the three response variables showed that (first model) the rut peak date was negatively affected by autumn rainfall, i. e. the more rain in autumn the earlier the rut peak next year. The other environmental or population variables did not show any significant effect in this first model, pointing to rainfall as the main driving factor of rut phenology among the variables studied. Autumn rainfall appears to be of central importance in Mediterranean environments to allow vegetation regrowth after the dry and hot summer [99, 100], which allow red deer to recover after the limiting season [35].

For roaring rate (second model), we found that autumn rainfall and mean temperature in midwinter associated with higher rate in the next rutting season, but the positive effect of temperature was lost when NDVI value in late spring was high. It may be explained by the vegetation growth, as mentioned above, since more rain in autumn and warmer temperatures in midwinter may favour an earlier spring, and therefore an earlier energy recovering, but when NDVI is high in late spring it means high resource availability for red deer, and therefore, the spring advancement effect loses its importance. The OSR had the greatest and positive effect in the model, increasing roaring rate, likely indicating higher intensity of signalling rutting behaviour when there were more rivals around [101]. Population density also had a high effect, likely because it increased rutting interactions.

The third model showed that OSR was also the greatest effect increasing the opportunity for sexual selection, likely because of the increase in male competition. The increase of the proportion of males and mate competition tend to enhance the variance in harem size or the proportion of unsuccessful males, both parameters positively related to the opportunity for sexual selection [73, 101, 102]. It may seem paradoxical, but our data have shown that roaring intensity and opportunity for sexual selection appear to be rather opposite outcomes. Roaring rates relate to male breeding activity either in intrasexual competition [70, 103] or in mate choice [71]. More roars in the area may indicate that more males are taking part in reproduction and hence females are more evenly distributed among males. Thus, higher opportunity for sexual selection occurs when harder environmental conditions increase the differences in mating success between the few successful males and the remaining males.

After controlling for population variables such as the OSR, the effect of environmental variables remained significant. Higher rainfall, temperature in midautumn, and NDVI in late spring, associated with reduced opportunity for sexual selection. As already mentioned, autumn rains favour the growth of vegetation after the limiting season in summer, allowing red deer to recover their nutritional status. Besides, when resources remain abundant in late spring (high NDVI), red deer can reach optimal physical conditions for reproduction. Thus, these years the rut activity occurs earlier and more intense, and sexual selection in the population appears to be less strong.

Along with their effects on the condition of individuals, environmental variables influence the distribution of resources during the rutting season, and resource distribution affects female distribution that, in turn, influences the opportunity for sexual selection [44–46, 84]. We estimated the opportunity for sexual selection by using de distribution of males and females [73]. In fact, in Doñana, spatial distribution of females and the distribution of reproductive success of males strongly depend on resource distribution [54, 55]. With patchy distribution of resources, red deer females in Doñana aggregate in few patches, which favours that males monopolyze relatively large harems [44, 54], thus increasing the opportunity for selection [46]. This is also consistent with the negative relationship between NDVI during June to July and the opportunity for sexual selection.

Regarding the effect of temperature, we do not have a straightforward explanation for the negative relationship between October temperature and opportunity for sexual selection in the next rutting season. October temperature may be critical because it occurs just after the rutting season and after the summer, which is the limiting season for deer in these habitats [35], and females are at the beginning of gestation. High temperatures usually mean drought events and poor conditions for vegetation in this area [38, 39]. Poor environmental conditions in late summer and early autum have been preiosuly related to a delay in red deer conceptions in Iberia [28]. In fact, when exploring our data (not shown), we found that October temperature seemed to be related to a delay in the roaring activity of the just finished rutting season (September), and the delay in the rutting season is normally associated with lower annual reproductive output (fertility and recruitment rates) in red deer [24, 41]. A bad year, with lower reproduction rates, has been previously related to better physical predisposition of red deer for the following year [96, 97]. Thus, October temperatures might relate to better conditions for many deer the next year and hence it might relax mating competition and opportunity for sexual selection. However, this is so far only a working hypothesis that deserves further research.

In the last decades, Doñana National Park has undergone changes due to climate change but also to indirect human pressure. The landscape has lost heterogeneity and its structure has been disturbed, mainly because of the decrease in the water table due to the intensive agriculture around it and the increase of water extraction for the urbanization of the adjacent area [56–59, 63–67]. This has produced a decrease in plant species richness and the replacement of herbaceous plants by woody ones [59–62, 66]. Moreover, prospects for climate change predict a decrease in rainfall in southwest Spain [29–32], which may also contribute to the reduction of herbaceous plants in Doñana, resulting in less resource availability and higher aggregation for herbivores.

Our results pointed to autumn rainfall (significant effect in the three models) as the main limiting factor in this type of Mediterranean ecosystems, and hence that the expected influence of climate change on Iberian red deer mating system should be mediated by rainfall and its effect in the water table and vegetation growth. In fact, these environmental conditions favourable for red deer seemed to be decreasing slightly over the years, as for autumn rainfall and winter temperature in Doñana. These conditions may delay or reduce resource availability and quality for red deer, which may prevent individuals from attaining the physical condition required for reproduction, and the rut may delay or even fail [27, 104]. A delay in conception date might produce a mismatch between actual births and optimal calving date, which may increase calf mortality [105, 106]. Moreover, nutritional stress during pregnancy and lactation has been shown to affect calf body mass and immune system which are related to their future survival and reproductive success [97, 104, 107, 108].

There are very few studies that relate environmental change and opportunity for sexual selection. It has been found that the intensity of sexual selection affected by local climatic variations in grey seals (*Halichoerus grypus*) by increasing the degree of polygyny in the population [109]. What we already know for red deer suggests that environmental changes may have consequences on selection and on population genetics, since variations in the distribution of females per male are related to differences in the genetic estimation of polygyny for Iberian red deer populations [44–46]. Thus, a higher variance in mating success could have long-term genetic consequences for red deer in Doñana. Studies on the transmission of genetic variability in Iberian red deer populations have shown that the level of intrasexual competition (i.e. degree of polygyny) was positively related to the transmission of genetic diversity via males, maybe due to the selective process by which successful males achieve matings [110]. The effective population size (Ne) is numerically reduced when polygyny is high [109]. However, sexual selection departs from the random reduction of breeding males that the estimation of Ne normally assumes [110]. Hence, higher opportunity for sexual selection may not be linked to an actual reduction of paternally transmitted genetic diversity, and even the opposite may be true, although this deserves further research.

In conclusion, this study from a Mediterranean area in Southern Europe provides new knowledge about the impact of the increasing drought events and temperature fluctuations derived from global change on red deer reproduction, affecting mating phenology, rutting behaviour, and the opportunity for sexual selection in the population. However, future consequences in the population genetics are unclear. The study also highlights the importance of considering environmental conditions, population dynamics and reproductive strategies when assessing the impact of climate change, since complex networks of responses to local climatic variations likely differ between species and locations.
